# Role of diffusion and reaction of the constituents in spreading of histone modification marks

**DOI:** 10.1371/journal.pcbi.1012235

**Published:** 2024-07-11

**Authors:** Vinoth Manivannan, Mandar M. Inamdar, Ranjith Padinhateeri

**Affiliations:** 1 Department of Biosciences and Bioengineering, Indian Institute of Technology Bombay, Mumbai, India; 2 Department of Civil Engineering, Indian Institute of Technology Bombay, Mumbai, India; 3 Sunita Sanghi Centre of Aging and Neurodegenerative Diseases, Indian Institute of Technology Bombay, Mumbai, India; Korea Institute for Advanced Study, KOREA, REPUBLIC OF

## Abstract

Cells switch genes ON or OFF by altering the state of chromatin via histone modifications at specific regulatory locations along the chromatin polymer. These gene regulation processes are carried out by a network of reactions in which the histone marks spread to neighboring regions with the help of enzymes. In the literature, this spreading has been studied as a purely kinetic, non-diffusive process considering the interactions between neighboring nucleosomes. In this work, we go beyond this framework and study the spreading of modifications using a reaction-diffusion (RD) model accounting for the diffusion of the constituents. We quantitatively segregate the modification profiles generated from kinetic and RD models. The diffusion and degradation of enzymes set a natural length scale for limiting the domain size of modification spreading, and the resulting enzyme limitation is inherent in our model. We also demonstrate the emergence of confined modification domains without the explicit requirement of a nucleation site. We explore polymer compaction effects on spreading and show that single-cell domains may differ from averaged profiles. We find that the modification profiles from our model are comparable with existing H3K9me3 data of *S. pombe*.

## Author summary

DNA is folded into chromatin by wrapping it around histone protein octamers, forming a string of nucleosome beads. The chemical modifications on histone proteins along the one-dimensional string form a significant part of the epigenetic code. This code determines whether a chromatin region is likely to be active or repressed. How the chemical modification patterns arise, and their stability along the histone proteins is a question of great interest. While some players in this process are known, how the physical process of diffusion and reaction of the enzymes collectively dictate the pattern formation is unknown. In this work, we simulate histone modification spreading accounting for explicit diffusion and reaction of the essential constituents. We show that the diffusion and degradation processes of the constituents limit the size of the modification domain. We also show how the availability of enzymes and the compaction of the chromatin affect the spreading of the histone modification.

## I. Introduction

Even though all cells in a multi-cellular organism have the same genetic code (DNA sequence), the different cell types in the organism, for example, neuronal cells and skin cells, behave very differently. This difference in function is achieved by encoding extra layers of information in the form of an epigenetic code [1, 2]. The significant players in encoding this epigenetic code are nucleosomes—147 bp of DNA wrapped around an octamer of histone proteins—positioned throughout the genome [3–5]. The functional genome is a chromatin polymer with nucleosome as its fundamental unit and other proteins helping in the assembly and disassembly of nucleosomes, alterations in chemical states of the histones, and 3D organization of DNA [6–11].

While the positions of nucleosomes along the DNA itself carry some epigenetic information, more information is encoded in the form of chemical modifications on the histones [2, 12–15]. For example, the 9^th^ amino acid lysine of histone H3 gets tri-methylated (H3K9me3) or acetylated (H3K9ac), and the presence of these marks along the chromatin polymer contour decides whether the local chromatin is inactive or active. Research in the last two decades has given us a basic understanding of how different chromatin marks affect the state of the local chromatin, including the 3D folding, accessibility of certain regions, and physiological state of the cell [16–22]. For instance, it is known that abnormal histone modifications can lead to various diseases, such as bone and brain cancer [23, 24]. While we understand the steady-state profiles of the histone modifications from ChIP-seq experiments [25, 26], very little is known about the kinetics of the formation of these profiles [27–29]. Therefore, it is important to understand these underlying mechanisms of histone modification pattern establishment.

Following DNA replication, the *de novo* assembly of chromatin states involves nucleation of histone modifications at specific target sites [30–33]. Experimental evidence shows that around the nucleation site, the modifications are able to spread to the neighboring regions and establish a stable pattern of modifications [32, 34, 35]. Specifically, H3K9me3 has served as a paradigm to study the spreading of histone modifications.

Hathaway *et al*. studied the spreading of H3K9me3 from Oct4 locus [36], and showed that the modification starts near a nucleation site and is confined to a limited region along the chromatin contour. In *Schizosaccharomyces pombe*, the mating type locus is repressed by the spreading of H3K9me3 [37, 38]. In this case, the histone methyltransferase complex (“reader/writer”) Clr4/Suv39h recognizes H3K9me3 and spreads the mark throughout the domain via RNAi mechanism [39, 40]. Recently there have been experiments that probe the heterochromatin assembly and the signaling pathways that govern it [41–43].

Many theoretical models were proposed to study the positive feedback of reader-writer enzymes in establishing chromatin domains [44–51]. Dodd *et al*. provided a model that investigates the heritability and bistability of acetylations and methylations at various locations along the genome [47]. Another model was proposed by Hodges *et al*. [52], where the spreading starts from the nucleation point and proceeds in a set of kinetic events, leading to a modification pattern that decays away from the nucleation point. Following these, many groups have explored different sets of kinetic events and non-equilibrium rules, studying the spreading and formation of histone modification patterns [53, 54]. The spreading along the linear dimension is the most natural event, given the beads on a string picture of chromatin. However, 3D spatial proximity between far-away nucleosomes could also be important, and recent papers have incorporated this into their models to explain the spreading [55–61].

If modifications can spread to all proximal nucleosomes in 3D, it is not clear exactly how a modification limits itself into domains of finite size. Some hypotheses have been proposed to address this question; they include having boundary elements that slow down the spreading [31, 54, 57, 62], looping mechanisms that relax chromatin quicker [55–60], the possibility of multiple intermediate states [63], the presence of large gaps between sliding nucleosomes [64–68], and enzyme limitation [61, 69].

Even though several papers study the spreading of histone modifications computationally, no model so far has explicitly considered the diffusion of the enzymes and RNA that are crucial in the spreading process. Existing models assume that the process is reaction-limited. However, two essential points concern this assumption: (i) Recent experiments have shown that the viscosity inside the nucleoplasm can be very high— orders of magnitude higher than water [70]. This implies that the diffusion can be very slow and can influence the effective time scale of the reactions [71]. (ii) Beyond the diffusion time scale, kinetic parameters such as the timescale of availability (or decay) of the enzymes/RNA-complexes can limit the spreading process. Since advances in experimental techniques are making it possible to perform single molecule dynamics in live cells that can measure diffusive properties [72], computational studies that investigate the contribution of diffusion to histone modification spreading can be useful.

In this paper, we examine the interplay between diffusion of enzymes/complexes and reaction-kinetic events in deciding the spreading of histone modifications. We propose a reaction-diffusion framework to study the establishment of modification domains following the nucleation events. We explicitly consider the diffusing factors and the production and decay of the constituents involved in the spreading process. We find that a basic kinetic model may not always explain the modification profile of an entire reaction-diffusion system. We also examine the effect of polymer folding and far-away contacts in our model.

## II. Model

### A. Reaction-Diffusion Model

We study the spreading of the histone modifications in a system consisting of a few kilobase (kb) of chromatin (*N*_*n*_ nucleosomes), enzymes that read/write histone modifications, and RNA. We use a particle-based reaction-diffusion model to understand the spreading of the histone modifications from a nucleation point. We choose a circle of radius *r* as our simulation space with *N* particles in it. The particles can be any one of the three types: Enzyme (*E*), RNA (*R*) or Enzyme-RNA complex (*C*). The diffusion of the particles in the system is simulated using Langevin dynamics (see Eq 1). Given the position of *i*^*th*^ particle at the *n*^*th*^ timestep (**r**_**i**_(*n*)), we update it for the next time step as follows:

$$\begin{array}{c} \tilde{r_{i}}\left(n+1\right)={\tilde{r}}_{i}\left(n\right)+\sqrt{2\tilde{D}}g \end{array}$$ (1)

where $\tilde{r_{i}}=(r_{i}/\sigma )$, *σ* = diameter of a particle, $\tilde{D}=\frac{\mu k_{B}T\Delta t}{\sigma^{2}}$ is the dimensionless diffusion constant, *μ* = mobility of particles, *k*_*B*_ = Boltzmann constant, *T* = Temperature, and **g** is a Gaussian random number with mean 0 and variance 1. We consider only the thermal kicks obeying the fluctuation-dissipation theorem [73]. We assume that all particles have the same size and diffusion constant ($10^{-3}\frac{\mu m^{2}}{s}$) for simplicity.

**Fig 1:**
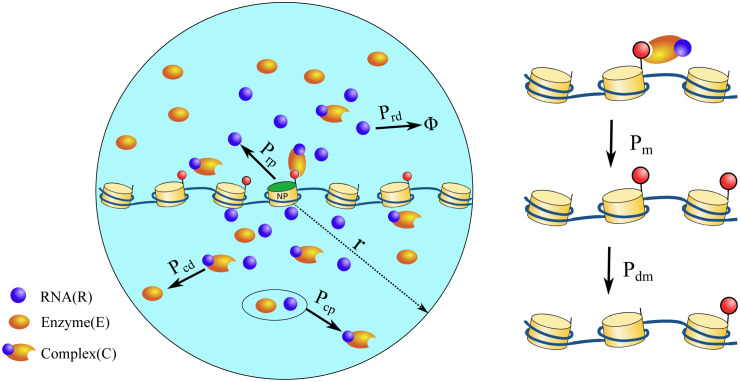
Schematic of the reaction-diffusion model in which each lattice site represents a nucleosome. Particles *R*, *E*, and *C* diffuse in the simulation space. *R* is produced only at the nucleation point (NP) with probability *P*_*rp*_. It can decay with probability *P*_*rd*_. When *R* and *E* come in proximity, they form *C* with probability *P*_*cp*_. *P*_*cd*_ controls the decay of *C* back to *E* (see Eqs 2 to 4). The *C* particles spread the modification to the nearest neighbor with probability *P*_*m*_ (see right-hand side). Each modified nucleosome can become unmodified with probability *P*_*dm*_.

Experimental evidence so far suggests that most of the DNA folding proteins (HP1, PRC, CTCF) bind at locations with specific histone modifications [74–76]. This implies that the spread of histone modifications, to some extent, would precede the 3D folding of chromatin. Hence, it is crucial to understand the kinetics of 1D spreading. Therefore, we consider chromatin as a 1D polymer in the first part of the work, assuming it is linear in the scale of a few nucleosomes and the most critical spreading events occur between the neighboring nucleosomes. In a later section (see section E.), we present results considering chromatin as a 2D polymer, examining the effect of the folding/compaction. In the 1D polymer picture, each nucleosome is a lattice point along the x-axis with its origin at the center of the circle (see Fig 1). The lattice spacing between neighboring nucleosomes is unity (*σ* units). For simplicity, each nucleosome can be either in Unmodified (U) or Modified (M) state. The nucleosome at the origin is considered the nucleation point (NP) and maintained at M state throughout the simulations. In fission yeast, there is evidence that the Clr4/Suv38h complex recognizes the existing modification in a nucleosome and propagates it to the neighboring nucleosomes [77]. The localization of these complexes is observed to be through RNAi mechanism [18, 38–40, 77]. RNAi mechanism involves the cleaving of RNAs (generated near the nucleation point) to form siRNAs, and then siRNAs become an integral part of these enzyme complexes. This is used by the cell as a localization mechanism for heterochromatic silencing [37]. To simulate the essence of the mechanism described above, we consider a simple scenario of RNA-like particles generating from the origin, which on collision with enzyme particles form enzyme-RNA complexes. The reactions involved in the model are:

$$\phi \overset{{\text{P}}_{\text{rp}}}{\underset{{\text{P}}_{\text{rd}}}{⇌}}\text{R}$$ (2)

$$\text{E}+\text{R}\overset{{\text{P}}_{\text{cp}}}{\to }\text{C}\overset{{\text{P}}_{\text{ed}}}{\to }\text{E}$$ (3)

$${{\text{N}}_{i}}^{\text{U}}\overset{{\text{P}}_{w}}{\underset{\text{C}}{\to }}{{\text{N}}_{i}}^{\text{M}}$$ (4)

$${{\text{N}}_{i}}^{\text{U}}+{{\text{N}}_{i\pm 1}}^{\text{M}}\overset{{\text{P}}_{m}}{\underset{\text{C}}{\to }}{{\text{N}}_{i}}^{\text{M}}$$ (5)

$${{\text{N}}_{i}}^{\text{M}}\overset{{\text{P}}_{\text{dm}}}{\to }{{\text{N}}_{i}}^{\text{U}}$$ (6)

RNA (*R*) is being produced only at the origin, with the probability *P*_*rp*_, and it can decay to *ϕ* with the probability *P*_*rd*_ anywhere in space as it diffuses. Even though the most straightforward interpretation of *R* is RNA, in a coarse-grained model, *R* represents the components of the RITS complex (e.g., Argonaute). *P*_*rd*_ represents the timescale the particle is available for the reaction. It can become unavailable by degradation or other mechanisms like chemical modifications and structural changes. Enzyme particles (*E*) are uniformly distributed in space at the start of the simulation. When *E* comes in contact with *R*, they form complex (*C*) particles with the probability *P*_*cp*_. Enzyme-RNA complex (*C*) particles can methylate a nucleosome with a probability *P*_*w*_. Also if one of the nucleosome’s neighbors (*N*_*i*±1_) is methylated, the probability of it getting methylated will be boosted by *P*_*m*_. Therefore, the effective probability of methylation will be, $P_{m}^{\text{eff}}=\delta_{C,1}(P_{w}+P_{m}\delta_{N_{i\pm 1},M})$. *δ*_*C*,1_ = 1 if *C* complex is present in the vicinity of a nucleosome, otherwise *δ*_*C*,1_ = 0. $\delta_{N_{i\pm 1},M}=1$ if either of neighboring nucleosomes is methylated, else $\delta_{N_{i\pm 1},M}=0$. The value of *P*_*w*_ is non-zero in only the results presented in section D.

### B. Model with only kinetic events

To compare our results of the reaction-diffusion model with existing models in the literature, we also simulate the dynamics of the spreading of histone modifications as a purely kinetic process, similar to the work done by Hodges *et al*. [52]. The nucleosomes are considered as a string of lattices (*j* ∈ [−30, 30]), which can stay either in a modified (M) or unmodified state (U). The lattice position “0” is assumed as the nucleation point, which is always maintained at the modified state. The kinetic events considered are: (i) Spreading: *k*^+^ represents the rate of addition of the marks to the lattices’ neighbors that are already modified. (ii) Turn-over: *k*^−^ represents the rate of removal of marks from any lattice. Given a set of parameters, the stochastic evolution of these states of the nucleosomes is simulated by a standard Gillespie Monte-Carlo algorithm [78–81].

## III. Results

Our aim is to study the role of diffusion and the reaction of enzymes and other necessary constituents in the spreading of histone modifications along the chromatin polymer chain. To do this, we consider a chromatin region having *N*_*n*_ nucleosomes with a nucleation site, RNA production, and reaction-diffusion mimicking RNAi mechanism along with enzyme diffusion and reactions.

We modeled the chromatin region as a string of a one-dimensional lattice of 61 nucleosomes (index, *i* ∈ [−30, 30]), where they can exist in the modified (e.g. methylated) or unmodified (e.g. unmethylated) state (see Fig 1). Since our interest is to study methylation spreading, for simplicity, we only consider methylated or unmethylated states. The simulation box contains *N*_*e*_ number of enzyme particles (E) that are uniformly distributed at the start of the simulation. During the simulation, RNA-like particles (*R*) are produced from a specific location (i = 0, also the nucleation point NP) on the chromatin (see Fig 1). The particles (*E* and *R*) diffuse in the simulation box, obeying Langevin dynamics. We have assumed reflecting boundary conditions for the simulation space. When they come in proximity (see Note A in S1 Text), they react with a probability to form enzyme-RNA complex (*C*) particles (see Fig 1 and section II.A). When *C* is near an unmodified nucleosome and if either of its neighbors is in the modified state, the nucleosome can get modified with a probability *P*_*m*_ (see Eq 5). Any modified nucleosome can become unmodified with a probability *P*_*dm*_ (see Eq 6). The value of *P*_*w*_ is zero throughout the manuscript, except for section D. During the simulation, we record the modification state of each nucleosome (methylated or unmethylated) and analyse it statistically.

### A. Enzyme-RNA complex and RNA reaction parameters influence the methylation profile

**Fig 2:**
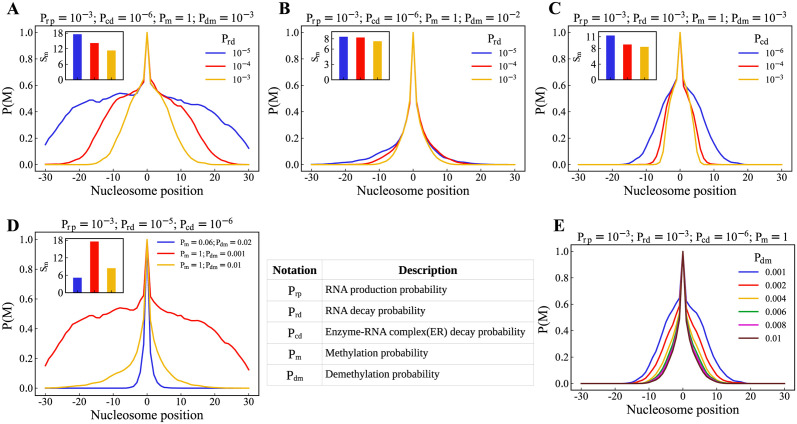
Effect of kinetic parameters on the methylation profile. Methylation profile compared by varying *P*_*rd*_ when *P*_*rp*_ = 10^−3^, *P*_*cd*_ = 10^−6^, *P*_*m*_ = 1 (A) *P*_*dm*_ = 10^−3^. (B) *P*_*dm*_ = 10^−2^. (C) Methylation profiles compared by varying *P*_*cd*_ when *P*_*rp*_ = 10^−3^, *P*_*rd*_ = 10^−3^, *P*_*m*_ = 1, *P*_*dm*_ = 10^−3^ (D) Methylation profiles by varying *P*_*m*_ and *P*_*dm*_. (E) Methylation profiles while varying *P*_*dm*_ from 10^−3^ to 10^−2^, where all the other parameters are constant.

Using an ensemble of snapshots from our simulation—modification of all nucleosomes—at the steady state, we compute the probability that the nucleosome at a given lattice site is methylated (*P*(*M*)). This site-dependent methylation probability would be referred to as the methylation profile in this manuscript. In this section, we vary the enzyme and RNA reaction parameters and investigate how the resulting methylation profile varies. The value of *P*_*w*_ = 0 as we are exploring only the spreading phenomenon. In Fig 2A, we show the methylation profile by varying only the RNA decay probability *P*_*rd*_ (see Eq 2) while keeping the remaining parameters constant. We see that the increase in *P*_*rd*_ from 10^−5^ to 10^−3^ significantly affects the spreading of the methyl marks, as inferred from the wider methylation profile. The spreading of the methyl marks is also quantified in the inset by plotting the standard deviation (*S*_*m*_) of the methylation profile for different sets of parameters,

$$\begin{array}{c} S_{m}={\left(\sum_{i=-\frac{N_{n}}{2}}^{\frac{N_{n}}{2}}i^{2}P_{i}-{\left(\sum_{i=-\frac{N_{n}}{2}}^{\frac{N_{n}}{2}}iP_{i}\right)}^{2}\right)}^{\frac{1}{2}} \end{array}$$ (7)

where *i* = the lattice position of the nucleosome, and *P*_*i*_ corresponds to the probability of modification (*P*(*M*)) of the lattice *i*. Fig 2B shows that at higher *P*_*dm*_ values, change in *P*_*rd*_ does not affect the spread of the methylation. Similarly, in Fig 2C, we plotted the methylation profile by varying the complex decay probability *P*_*cd*_ (see Eq 4). As *P*_*cd*_ decreases, the spreading increases in the case of *P*_*dm*_ = 10^−3^. In contrast, it remains unchanged when *P*_*dm*_ = 10^−2^ (see S1 Fig). This implies that *P*_*dm*_ = 10^−2^ dominates the effects of *P*_*rd*_ and *P*_*cd*_. Meanwhile, when *P*_*dm*_ = 10^−3^, we can see a competition between these values to spread the methyl marks. A phase diagram of the time evolution of modifications in the nucleosomes and the resulting profile is shown in S2 and S3 Figs, as we vary *P*_*rd*_ and *P*_*cd*_.

The enzyme-mediated modification reactions of the nucleosomes occur stochastically at certain rates. The reactions have two steps. In the first step, the reactants diffuse and reach near the nucleosomes. Even after reaching the proximity of nucleosomes, the reactions are not immediate; it has a stochastic nature and hence occur with a methylation probability *P*_*m*_ (see Eq 5) in our simulations. The demethylation reaction is accounted for by a probability *P*_*dm*_. In Fig 2D, we compare the methylation profile from three sets of simulations that have the same reaction parameters for R and C (*P*_*rp*_ and *P*_*rd*_, *P*_*cp*_ and *P*_*cd*_), but different *P*_*m*_ and *P*_*dm*_ (See subfigures’ title for the exact values of the parameters). We see that increasing *P*_*m*_ from 0.06 to 1 results in a slightly wider methylation profile, whereas decreasing *P*_*dm*_ from 10^−2^ to 10^−3^ results in the spread of the methyl marks to the extremities of the lattice. We vary only *P*_*dm*_ in Fig 2E while keeping all the other parameters constant. The methylation profile gets narrower as we increase *P*_*dm*_.

We also show the effects of the reaction parameters for the production and decay of *R* and *C* (*P*_*rp*_, *P*_*rd*_, *P*_*cp*_, *P*_*cd*_) in the spatial distribution of *R* and *C*. The particles diffuse and react in the 2D simulation space. To quantify the spatial distribution of RNA, we compute the number density of RNA (see S4 Fig) of concentric circles of increasing radii *r*_*i*_ as,

$$\rho_{i}=\frac{N_{R}^{i}}{\pi (r_{i+1}^{2}-r_{i}^{2})}$$

where, $N_{R}^{i}$ is the number of RNA in the *i*^*th*^ shell and *r*_*i*_ is the radius of the *i*^*th*^ circle.

**Fig 3:**
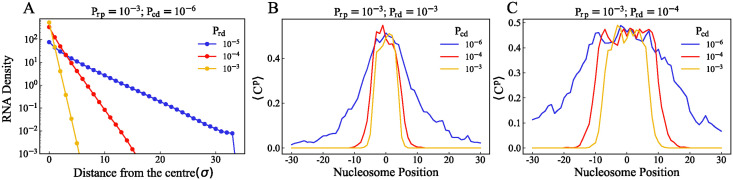
Spatial distribution of *R* and *C* at the steady state. (A) Number density of R present in the *i*^*th*^ concentric shell (*ρ*_*i*_) for different values of *P*_*rd*_, *i* = 0 refers to the center of the simulation space (see text) when *P*_*rp*_ = 10^−3^. (B) Average number of C particles proximal to the nucleosome position (〈*C*^*p*^〉) is plotted against nucleosome position for different *P*_*cd*_ values when *P*_*rd*_ = 10^−3^. (C) when *P*_*rd*_ = 10^−4^.

Fig 3A shows the number density of RNA in the *i*^*th*^ shell, calculated when *P*_*rp*_ = 10^−3^. The RNA profile gets narrower as we increase *P*_*rd*_. Also, we can notice that there is no difference in the RNA profile by changing the value of *P*_*rp*_ to 10^−4^ (see S5 Fig). Therefore, we have kept the value of *P*_*rp*_ = 10^−3^ throughout this paper. The width of the profile represents the length scale (*l*_*r*_) beyond which RNA cannot be found and can be estimated as $l_{r}∼\sqrt{D_{r}/P_{rd}}$, where *D*_*r*_ is the diffusion coefficient of the RNA particles. We have kept the value of *D*_*r*_ = 2 × 10^−4^ throughout this manuscript. However, the methylation profiles corresponding to different values of *D*_*r*_ are presented in S6 Fig.

When E and R particles come in proximity, the enzyme-RNA complex (*C*) is formed with a probability *P*_*cp*_ (see Eq 3). The mean number of *C* particles proximal to the nucleosomes is determined by the RNA length scale (*l*_*r*_) and the probabilities *P*_*cp*_ and *P*_*cd*_. Since the meeting of the E and R is less likely, we have assumed the formation of *C* to be diffusion-limited, by keeping the *P*_*cp*_ = 1 in the results we present. Fig 3B shows the mean number of complex particles proximal to the nucleosomes for different *P*_*cd*_ values, when *P*_*rd*_ = 10^−3^. At higher *P*_*cd*_, the complex particles are more localized to the nucleation point. The same quantity for simulations with *P*_*rd*_ = 10^−4^ is plotted in Fig 3C. We can see that profiles of the complex particles in Fig 3C are wider than the corresponding profiles in Fig 3B, which is due to the higher *P*_*rd*_ value of the latter.

### B. Enzyme limitation influences the methylation profile

Conventional kinetic models assume enzyme supply to be infinite, and the reactions go on at a constant rate forever. Typically, in cells, the amount of protein present is finite. Accounting for that is referred to as enzyme limitation. Since our model explicitly considers a finite number of diffusing protein particles, enzyme limitation is naturally incorporated into our model. We investigated enzyme limitation in two ways: (i) varying the radius of the system (area of the simulation region) fixing the number of enzyme particles (*E* and *C*) constant, effectively varying the concentration of the enzymes, and (ii) changing the total number of the constituents (proteins and RNA), fixing the area of the simulation region constant.

**Fig 4:**
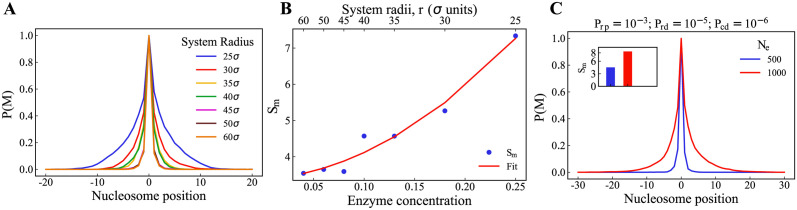
Effect of the total concentration of the constituents on the average methylation profile. The spreading is considerable with an increase in density. (A) The methylation profile is plotted across the lattice positions for systems of various radii (signifying concentration of the system). (B) The standard deviation of the methylation profile(*S*_*m*_) vs total concentration of the system, (C) Comparison of *N*_*e*_ = 500 and *N*_*e*_ = 1000 when *P*_*rp*_ = 10^−3^.

For case (i), we simulated a 41-nucleosome lattice with 500 enzyme particles (*N*_*e*_) for a given set of reaction parameters and varied only the radius of the simulation region, as mentioned above. In Fig 4A, we show the methylation profiles for the system radii ranging from 25*σ* to 60*σ*. The spread of the methyl marks is dependent on the concentration of the particles, which is inversely related to the radius of the simulation region.

In Fig 4B, the standard deviation *S*_*m*_ (see Eq 7) is plotted against the concentration of enzyme in the system (concentration = *N*_*e*_/*πr*^2^), where *N*_*e*_ is the number of enzyme particles in the system and *r* is the radius of the system. We have fit the data with the quadratic equation *y* = *ax*^2^ + *bx* + *c*, where x = enzyme concentration and the fitting parameters a, b, and c are 54.15, 2.05, and 3.37, respectively. It increases with increasing enzyme concentration (i.e., reduction of radii). The resulting concentration profile for the complex is shown in S7 Fig. The spreading is close to zero at any concentration below 0.1. This elucidates the importance of the local concentration of the enzymes in the spreading. For case (ii), we compared the methylation profiles for the number of enzyme particles (*N*_*e*_) as 500 and 1000, which is shown in Fig 4C. Despite having the same reaction parameters, the *N*_*e*_ = 1000 particle system has a wider methylation profile than the *N*_*e*_ = 500 particle system.

### C. Comparison of the reaction-diffusion model with a reaction-only model

Models with only kinetics of modification reactions—without diffusion—have been employed to describe the spreading of the modifications. The basic version of such models contains the spreading event of modification from a nucleosome to its neighbor with a rate *k*^+^, and the removal of modification with a rate *k*^−^. Such a method was implemented by Hodges *et al*. [52] to predict the modification profile. Similar methods were also used by other groups and the model was extended to incorporate more details into the kinetic events [53, 54]. However, these papers do not consider the effect of diffusion of enzymes.

**Fig 5:**
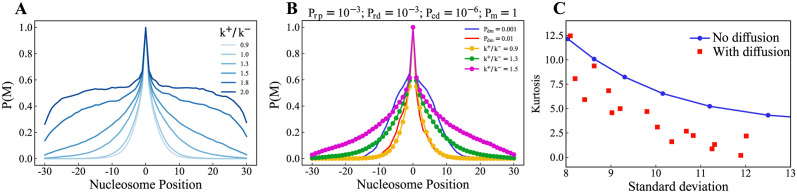
Comparison of methylation profile from the reaction-diffusion particle model to the KMC model. (A) The Methylation profile from the KMC model varied with the parameters. (B) Comparison of the methylation profiles from RD Model with that of KMC. (C) Kurtosis vs Standard deviation of the simulations with and without diffusion.

To understand the effect of diffusion, we compare the results from our reaction-diffusion model with a modified version (see S8 Fig) of the basic reactions-only model [52]. In this modified kinetic model, the nucleation point is always maintained in the methylated state (see section II.B). Fig 5A shows the methylation profile generated by the Gillespie method for various parameters of $K=\frac{k^{+}}{k^{-}}$, which is the ratio of the rate of the spreading to the rate of removal. The methylation profile widens as we increase *K*. In Fig 5B, we compare the methylation profiles generated from the diffusion-limited case (*P*_*m*_ = 1) of the reaction-diffusion model to the KMC results. We see that the methylation profile when *P*_*dm*_ = 0.01 matches the methylation profile from the KMC model of *K* = 0.9. However, the methylation profile generated for *P*_*dm*_ = 0.001 is not comparable with either of the two profiles (*K* = 1.3, *K* = 1.5). It suggests that all the profiles generated through the reaction-diffusion model are not reproducible through a basic single-step reactions-only model; incorporating diffusion alters the nature of the profile. However, there could be different types of multi-step processes that could also change the profile.

To compare the profiles from the two different models, we calculated the kurtosis [82] and standard deviation for each profile, as these moments characterize the nature of the distribution. In Fig 5C, kurtosis values of the methylation profiles are plotted against the standard deviation. We can see that the curve which corresponds to the KMC simulations (No diffusion) stay above the trend of the simulations where the diffusion is accounted for (squared red dots). This implies that larger confined domains (high values of standard deviation) with lower kurtosis can be explained only when considering the constituents’ diffusion.

To test whether the modification profile arising from the RD model can be captured in an effective kinetic model, we have made the spreading rate *k*^+^ to be space-dependent ($k^{+}(i)=k_{0}e^{-\frac{\left|i\right|}{l_{d}}}$). Here, *l*_*d*_ refers to the length scale of the spread of the enzyme complexes from diffusion and reaction. This is introduced here as an extra parameter; in our RD simulations, this naturally arises from the more microscopic (or finer) reaction parameters. These simulations at higher values of *K* (= $\frac{k_{0}}{k^{-}}$) also produce profiles similar to the results of the RD model (see S9 Fig).

### D. Confined Methylation profile with a peak can emerge in the absence of a nucleation point

**Fig 6:**
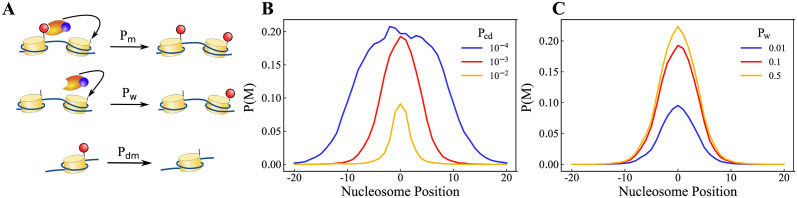
Emergence of nucleation point as a result of the RNA-based mechanism (Model without a nucleation point and a modified probability). (A)Schematic of the nucleosome modification reactions. When Complex particles are in the proximity of unmodified nucleosomes, they can put a mark with probability *P*_*w*_, and if one of its neighbors is modified, this probability will be boosted by *P*_*m*_. (B) Modification profile comparison while varying the rate of complex decay(*P*_*cd*_). (C) Modification profile for different writer probability (*P*_*w*_).

To test the role of the nucleation point, we check a modified version of the reaction-diffusion model (see section II.) with two changes: (i) Absence of nucleation point—i.e., the nucleosome at i = 0 is like all the other nucleosomes, and it is not constantly in a methylated state, (ii) The enzyme-RNA complex can add methyl marks, with a probability *P*_*w*_, even if the neighbors are not methylated. This is equivalent to an enzyme diffusing and stochastically methylating a random nucleosome, which is in addition to the probability of spreading (*P*_*m*_) to the neighbors by methylated nucleosomes (see Fig 6A). Fig 6B shows the plots of methylation profile where kinetic parameters are kept constant, and the curves correspond to different *P*_*cd*_ values ranging from 10^−4^ to 10^−2^. Similar to the results presented in Fig 2C, the methylation profile corresponding to the lower *P*_*cd*_ value is wider. Since the nucleosome at *i*=0 is not methylated at all times, the shape of the profile is not the same as that of Fig 2. However, note that there is a peak emerging at *i*=0. Fig 6C shows the methylation profiles for different *P*_*w*_ values. Interestingly, as *P*_*w*_ increases, the peak height of the methylation profile increases.

The emergence of the peak at the center of the simulation region without the necessity of a nucleation point can be explained by the RNA particles being produced there. As a result, the enzyme-RNA complex profile peaks around the center, which in turn determines the methylation profile. This suggests that the nucleation points, although meant to be inherited by the daughter cells from the mother cells, can also be a by-product of gene expression from a specific loci. This is achieved by considering the “writer” enzyme property along with “reader-writer” enzymes.

### E. Chromatin polymer folding affects the modification profile and comparison with experimental data

So far, we have assumed the chromatin as a 1-dimensional string. Nevertheless, in cells, chromatin is folded and packed. Each nucleosome will have a few other nucleosomes in proximity (close contact) beyond the two neighbors along the polymer backbone. Some of the recent papers have studied 1-dimensional chromatin but accounted for non-neighboring methylation spreading by modifiying the spreading rates [60, 61, 69]. Some other papers have considered the explicit polymer nature for studying the spread of the modifications [48, 57, 83–86]. Going beyond these models, in this section, we account for chromatin’s folded polymer nature as well as explicit diffusion of constituents and reactions with the nucleosomes. We study the effect of far-away contacts in spreading modifications, and for simplicity, we restrict ourselves to 2D polymer networks.

**Fig 7:**
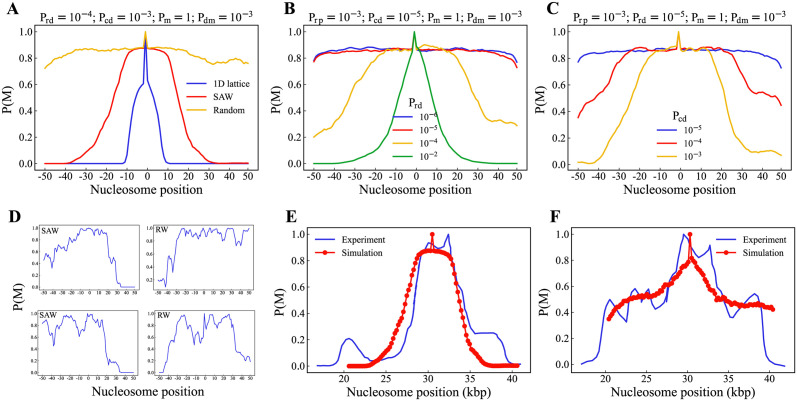
Chromatin compaction engages the spreading. (A)Average Modification profile compared between polymers of different compaction, Average of methylation profile over an ensemble varying with kinetic parameters (B) *P*_*rd*_, (C)*P*_*cd*_, (D) Individual modification profiles from frozen configurations of SAW polymer and random walk polymer, (E) H3K9me3 profile of *hht*2^*G*13*D*^ mutant of the mating-type loci in *S.Pombe* compared with the profile generated from an ensmeble of SAW polymers, (F) H3K9me3 profile of Wildtype of the above mentioned genome compared with profile generated from random walk polymer.

We simulate a fragment of chromatin as a 100-bead self-avoiding walk and random walk polymers separately (see Note B in S1 Text). We take an ensemble of configurations from those simulations. For each of these configurations, we simulate the diffusion and reaction of all the constituents (R, E and C) similar to what is done for the linear polymer, and the resulting modification profile is recorded (see section. II.). A few such individual methylation profiles from spreading along the self-avoiding walk polymer configurations are shown in Fig 7D. Note that the individual profiles generated from spreading along the individual configurations do not typically have a highly peaked and symmetrically decaying profile. This implies that we can expect similar profiles whenever the spreading time is less than the polymer relaxation time. Even though the individual profiles do not have a clearly confined domain of modified nucleosomes when we average over an ensemble of configurations, a different profile emerges (see Fig 7B), in which we can see a confined modification domain. In Fig 7A, we have compared the profiles generated from a linear lattice with that of polymers with two different degrees of compaction (self-avoiding walk and random walk) while keeping all the other kinetic parameters constant. We can clearly see that the modification spreads more on a random walk polymer than on a self-avoiding polymer. This is expected since each nucleosome in a random walk polymer have many more neighbors. Also, The size of the modification domain decreases as we increase the kinetic parameters *P*_*rd*_ (see Fig 7B) and *P*_*cd*_ (see Fig 7C). The results presented in Fig 7B and 7C are all ensemble-averaged quantities from random polymer configurations.

To test whether our simulation results are biologically realistic, we compare them with the experimental data from the Dipiazza *et al*. [77]. They study the spreading mechanism of H3K9me3 from mating type loci (roughly 20kbp) in *S.Pombe* using a dominant-negative mutant *hht*2^*G*13*D*^. They establish that the H3K9me3 levels from ChIP-seq are reduced when they use this mutant. We find that the H3K9me3 profile generated from this mutant matches with SAW configurations with reaction parameters *P*_*rd*_ = 10^−4^, *P*_*cd*_ = 10^−3^ and *P*_*dm*_ = 10^−3^ (see Fig 7E). Similarly, the H3K9me3 profile of wild-type is comparable with the profile generated from polymers configurations having higher compaction (random walk) with reaction parameters *P*_*rd*_ = 10^−4^, *P*_*cd*_ = 10^−4^ and *P*_*dm*_ = 10^−3^ (see Fig 7F). This indicates that the profile we obtained in our simulations are realistic. The difference between the mutant and wild-type could be the level of compaction of the domains or the concentration profile of the constituents around the region. Although experiments can currently verify the contact probability of the said regions, the varying concentration profile is an area for future research. However, the interplay between these two factors determines the epigenomic state of a given genomic region.

## IV. Discussion and conclusion

In this paper, we propose a reaction-diffusion framework to understand the spreading of histone modifications along the nucleosomes. The key difference in our model from other models in the literature is the explicit accounting of the diffusion of proteins/RNAs and mimicking of the RNAi mechanism. We consider a flux of RNA-like (R) particles produced from the nucleation point. These particles diffuse and meet with uniformly distributed diffusing enzyme-like (*E*) particles and then catalyze the propagation of the spreading in our model.

Our main results are as follows: We demonstrate the possibility that the diffusion of the constituents can play an essential role in deciding the modification pattern. First, we investigate how the diffusion coefficient and the decay rate of enzyme/protein/RNA constituents set a length scale beyond which the modification cannot spread. This naturally limits the domain size. Second, the results suggest that the distribution of the modification pattern depends on the diffusive nature of the constituents and is different from that of a basic kinetic model. Another way of including this diffusive aspect is considering a spatially dependent rate of spreading, in which a diffusive length scale exists. We also simulate how the RNAi mechanism would lead to the emergence of the peak, and we do not have to maintain a nucleation point where a modification-state is consistently maintained with high probability. This suggests that the nucleation sites, which are assumed to be inherited from mother cells, can just be active genes or regions. Some models claim that a nucleation site is required to maintain confined domains, while others suggest a long-range loop-based spreading mechanism mainly to explain the profile [55–58]. It seems more plausible for the switch to be a diffusion-limited mechanism inside the nucleus.

The parameters that control the instantaneous number of these particles are the production and decay probabilities of RNA and the enzyme complex (namely, *P*_*rp*_, *P*_*rd*_, *P*_*cp*_, and *P*_*cd*_). The change in *P*_*rp*_ does not affect the length scale of RNA (see S5 Fig). The modification profile gets wider with the decrease in RNA decay probability (*P*_*rd*_) as well as complex decay probability (*P*_*cd*_). However, this change is not observed in the regime of higher demethylation probability (*P*_*dm*_ = 0.01). Another factor influencing the modification profile in our model is the availability of enzymes. Enzyme limitation has been considered in recent papers by incorporating it into the parameters of kinetic rates [69]. However, enzyme-limitation arises naturally in this model since a given number of enzyme-like particles are part of our model. We varied this enzyme concentration and observed the modification profile getting wider as we increase the concentration. The final aspect we present is the role of chromatin compaction in the spreading of histone marks [48, 57, 83–86]. We examine how different degrees of compaction can dictate the modification profile among an ensemble of cells. We compare our model predictions with the H3K9me3 ChIP-seq profiles reported in mating-type loci of *S.pombe*.

In principle, there can be other models (with more kinetic rate parameters) that can give rise to results similar to ours. The extra parameters could be effective rates that incorporate the waiting time due to diffusion or other events such as intermediate states in the modification process. However, our aim here is to explicitly account for the diffusion of the constituents. Accounting for proteins explicitly has several advantages: (i) It is a fact that the proteins are diffusing in the nucleoplasm, which is highly viscous and crowded. Hence, the extent of diffusion can play an essential role in spreading. (ii) The spatial distribution of particles is related to other known events like phase separation etc. Such a model with explicit diffusion can be extended in the future to account for phenomena like phase separation of proteins. For example, ubiquitination is argued to be related to phase separation of readers/writers [87]. Since no one has explicitly examined the role of diffusion and the resulting spatial distribution, investigating this problem as a reaction-diffusion system is crucial.

The phenomenon we discuss in the paper is the dynamic maintenance of the steady-state concentration of the complexes involved. An alternative scenario could be liquid-liquid phase separation of the constituents involved. There is evidence of heterochromatin liquid droplets across many species [88–90]. However, to our knowledge, no one has reported microphase separation of these Clr4-like constituents *in vivo*. Even if phase separation drives the spreading, decaying profiles with a peak is not what one would expect from that scenario. Another possibility is that the histone itself can diffuse during DNA replication which has been studied in the recent paper [91] in the context of histone modifications inheritance. These are other things that can be further studied in the future, along with the aspects discussed in our model.

Since most biological processes are determined by an interplay between chromatin organization and reaction-diffusion of proteins, we hope that our model will serve as a starting point in exploring several questions in this direction. The model can be extended to study cooperative (or anti-cooperative) interaction between many different reader/writer enzymes and modifications and the role of many different proteins. Given that many proteins show liquid-like condensation behavior and such condensation is relevant for gene regulation, one has to go beyond the kinetic models and incorporate the reaction-diffusion of proteins into a chromatin model to predict statics and dynamics of the crucial biological mechanisms.

## Supporting information

S1 Fig Effect of *P*_*cd*_ in the modification profile, corresponding to the parameters when *P*_*dm*_ = 10^−2^. We see no difference between the different profiles, since *P*_*dm*_ dominates the effect of complex decay kinetics.
(EPS)

S2 Fig Phase diagram of time evolution of modification state of nucleosome lattice as we vary the RNA decay parameter (*P*_*rd*_) and Enzyme-complex decay parameter (*P*_*cd*_). Each individual plot corresponds to a specific *P*_*cd*_ and *P*_*rd*_ value while all the other parameters are kept constant.
(PNG)

S3 Fig Phase diagram of modification profile as we vary the RNA decay parameter (*P*_*rd*_) and Enzyme-complex decay parameter (*P*_*cd*_). Each individual plot shows the steady-state average modification profile corresponding to the parameters mentioned in the axes.
(EPS)

S4 Fig Schematic of RNA density(*ρ*_*i*_) of *i*^*th*^ shell. It is the ratio of the number of RNA particles in *i*^*th*^ shell (the blue strip of radius *dr*) to the area of the corresponding shell.
(EPS)

S5 Fig Spatial distribution of *R* at the steady state. Variation in number density of R present in the *i*^*th*^ concentric shell (*ρ*_*i*_) with *P*_*rd*_, 0 starting from the center(see text) when *P*_*rp*_ = 10^−4^. Despite of ten-fold smaller *P*_*rp*_, the RNA density remains similar to Fig 3A.
(EPS)

S6 Fig Methylation profile when we vary the mobility term (*D*_*r*_) in the brownian motion. As we discussed in the main text, the diffusion coefficient and the kinetic parameters together decide the length scale of the modification domains. But we have performed simulations with a constant value of *D*_*r*_ to study the effect of other constituents. However, with a different *D*_*r*_, we would get different sized domains. We have chosen the value *D*_*r*_ = 2 × 10^−4^, assuming the particle size to be around 5–10 nm, which is biologically relevant.
(EPS)

S7 Fig Average proximity of enzyme-complex(*C*^*p*^ plotted against nucleosome position). The different curves correspond to respective system radius. We employed this varied system radius to account for enzyme limitation.
(EPS)

S8 Fig Schematic of Kinetic Monte-Carlo method (KMC). The nucleation point (*NP*) is kept methylated at all times. The modified nucleosomes can spread their modifications to their neighbours with the rate *K*^+^ and any modified nucleosome can become unmodified with the rate *K*^−^.
(EPS)

S9 FigResults of Kinetic Monte-Carlo method (KMC) where the rate of spreading *k*^+^ decays exponentially ($k^{+}(i)=k_{0}e^{-\frac{\left|i\right|}{l_{d}}}$) as it moves away from the nucleation point.A) The steady state modification profile of KMC simulations, as we increase the value of *K*, which is equal to the ratio of $\frac{k_{0}}{k^{-}}$, B) Kurtosis vs standard deviation of the profiles generated by basic KMC, reaction-diffusion and KMC with exponential decay rate of spreading models. We can see that the lower kurtosis for a given value of standard deviation is achieved only when one considers the effect of diffusion.(EPS)

S1 Text Contains all the information mean proximity calculation (Note A) and the polymer dynamics simulation (Note B) used in Fig 7. (PDF)
