# Supplementary material for: Role of diffusion and reaction of the constituents in spreading of histone modification marks
Source: PLoS Comput Biol. 2024 Jul 11;20(7):e1012235. doi: 10.1371/journal.pcbi.1012235 (PMC11265668; doi:10.1371/journal.pcbi.1012235)
Supplement: S1 Text — (PDF) [file pcbi.1012235.s010.pdf]

SUPPLEMENTARY INFORMATION
Role of diffusion and reaction of the constituents in spreading of histone modification marks

Vinoth Manivannan,[1] Mandar M. Inamdar,[2] and Ranjith Padinhateeri*[1,3]

[1]*Department of Biosciences and Bioengineering, Indian Institute of Technology Bombay, Mumbai 400076, India*
[2]*Department of Civil Engineering, Indian Institute of Technology Bombay, Mumbai 400076, India*
[3]*Sunita Sanghi Centre of Aging and Neurodegenerative Diseases, Indian Institute of Technology Bombay, Mumbai, 400076, India*

* ranjithp@iitb.ac.in

Note A. Mean proximity calculation

A particle is considered proximate to a nucleosome lattice if it lies in the rectangular area formed by the position of the lattice, $y = 2$, and $y = -2$ line. The average number of particles in the area through the time steps is mentioned as "Mean proximity". For example, $< C^p >$ denotes the average number of complex particles found near a given nucleosome lattice.

Note B. Polymer simulation

We considered chromatin as a 2D bead-springs polymer chain of N beads. Each bead in this system corresponds to a nucleosome. We then equilibrated this polymer as a self-avoiding walk and random walk separately. The total energy of the self-avoiding walk polymer is given by,

$$E = \sum_{i=1}^{N-1} E_i^s + \sum_{i,jj>i} E_{LJ}(|r_{ij}|) \tag{1}$$

The beads in this polymer are connected through a harmonic spring, whose potential is given by,

$$E_i^s = \frac{K_s}{2}(|\vec{r_i} - \vec{r}_{i+1}| - r_0)^2 \tag{2}$$

where, $\vec{r_i}$ is the position of the ith beads, r_0 is the equilibrium bond length and K_s is the spring constant

All other non-bonded beads interact with LJ potential to achieve volume exclusion.

$$E_{LJ}(r_{ij}) = \begin{cases} 4\epsilon\left[\left(\frac{\sigma}{r_{ij}}\right)^{12} - \left(\frac{\sigma}{r_{ij}}\right)^{6}\right] & r_{ij} < 2^{1/6}\sigma, \\ 0 & r_{ij} \geqslant 2^{1/6}\sigma. \end{cases} \tag{3}$$

where,σ is the size of a particle, ϵ is the strength of the interaction, and r_{ij} is the inter-particle distance.

Then, we performed Brownian dynamics simulations of this model. After the equilibration, we took 100 timeframes such that each frame is 10^5 timesteps apart from the previous frame. The corresponding position information of the beads were used as frozen configurations for simulating the compaction effects in the reaction-diffusion model. In the case of random walk polymer, the total energy in the system was only the spring potential. Similar procedure was carried out to obtain an ensemble of frozen configurations of random walk polymers.
